# The Assessment of Meloxicam Phototoxicity in Human Normal Skin Cells: In Vitro Studies on Dermal Fibroblasts and Epidermal Melanocytes

**DOI:** 10.3390/molecules27134215

**Published:** 2022-06-30

**Authors:** Marta Karkoszka, Jakub Rok, Klaudia Banach, Justyna Kowalska, Zuzanna Rzepka, Dorota Wrześniok

**Affiliations:** Department of Pharmaceutical Chemistry, Faculty of Pharmaceutical Sciences in Sosnowiec, Medical University of Silesia, Jagiellońska 4, 41-200 Sosnowiec, Poland; d200971@365.sum.edu.pl (M.K.); jrok@sum.edu.pl (J.R.); kbanach@sum.edu.pl (K.B.); jkowalska@sum.edu.pl (J.K.); zrzepka@sum.edu.pl (Z.R.)

**Keywords:** meloxicam, phototoxicity, human normal skin cells, melanocytes, fibroblasts

## Abstract

Meloxicam (MLX), which belongs to the oxicam nonsteroidal anti-inflammatory drug derivatives, is an inhibitor of the cyclooxygenase-2 (COX-2) enzyme. Cutaneous adverse effects caused by interaction between UVA radiation and exogenous factors can manifest as phototoxic reactions. Phototoxicity may be a reason for the accumulation of genetic and molecular changes in long-lived cells with low proliferation potential, leading to tumor development. There are several potentially phototoxic drugs, the active component of which is meloxicam. The research aimed to evaluate the influence of MLX and UVAR on skin cells—fibroblasts and melanocytes homeostasis. The obtained results indicated that co-treatment with MLX and UVAR inhibited skin cell proliferation, proportionally to the drug concentration. The observation was confirmed by cytometric analysis of the cell number and viability. The phototoxic effect of MLX was revealed in morphological changes. It was stated that MLX with UVAR lowered the mitochondrial transmembrane potential and changed the cell cycle profile. Additionally, MLX and UVAR caused the disruption of redox homeostasis by lowering the intracellular level of reduced thiols. The presented study revealed that the phototoxic activity of MLX is associated with oxidative stress induction and disruptions in cell homeostasis. The differences in the phototoxic effects of MLX at the cellular level may be related to the different content of melanin pigments.

## 1. Introduction

The skin is considered to be the main barrier that protects the human body against many extrinsic harmful factors [1]. Epidermal cells, due to their location, are vulnerable to the negative impact of both physical and chemical factors, which may result in an increased frequency of oncogenic mutations [2,3]. Ultraviolet radiation (UVR) plays a key role in maintaining the human body’s homeostasis by regulating melanin synthesis and the concentration of vitamin D3, which is necessary for the proper functioning of the immune and skeletal systems. UVR type A (UVAR) represents approximately 95% of the whole UVR reaching the Earth’s surface, and its wavelength is from 320 nm to 380 nm. UVAR is able to penetrate deep layers of the skin. UVB radiation, which ranges from 280 to 320 nm, represents only about 5% of total UV energy and affects mainly the outer layers of the epidermis. UVC radiation is almost completely absorbed through the ozone layer of the Earth and has no effect on a person [2,4]. The combination of skin exposure to ultraviolet radiation and photoreactive xenobiotics may cause the occurrence of adverse skin reactions resulting in cell damage and accumulation of genetic and molecular changes, which may consequently lead to tumor development, especially in long-lived cells with low proliferation potential [3,4,5]. 

One of the most frequently occurring adverse drug reactions (ADRs) is cutaneous ADRs, which are recognized as major health problems worldwide. Drug-induced photosensitivity reactions are based on the mechanism of action of a given drug, the administered dose, and genetic predisposition, which may result in serious, irreversible skin cells damage [6,7]. Taking presented facts into consideration, skin ADRs usually require additional medical care, e.g., hospitalization and generate significant costs for health care systems [8]. Moreover, the skin-related ADRs are also the cause of patients’ comfort decreasing and may lead to limitations in pharmacotherapy effectiveness [1]. 

The most common types of drug-induced adverse skin reactions are phototoxicity and photoallergy. They appear as a result of the simultaneous interaction between the UVR and a chemical agent, which can be administered topically or systematically [9]. 

Phototoxic reactions are characterized by their occurrence after a short time (minutes to a few hours) after sun exposure, their appearance after the first exposure to a photosensitive agent, their dose-dependent effect and the necessity of high photosensitizer concentration [8]. 

In the phototoxic reactions, there is often formed reactive oxygen species (ROS) e.g., singlet oxygen, hydrogen peroxide, superoxide anion, and hydroxyl radical, which can induce oxidative cell damage [2,10,11,12]. ROS leads to direct cellular damage such as membrane destruction or intracellular component injuries, i.e., mitochondria, nucleus, or lysosomes [13]. Visible skin changes that occur during a phototoxic reaction resemble sunburn [14]. 

Meloxicam (MLX) belongs to oxicam nonsteroidal anti-inflammatory drugs (NSAIDs) derivatives. The drug is a selective inhibitor of cyclooxygenase-2 (COX-2), an immediate-response enzyme that catalyzes the synthesis of prostaglandins in response to various stress factors. The main effects of the drug are analgesic, antipyretic and anti-inflammatory properties [15,16]. 

Nowadays, MLX is widely used for the long-term treatment of musculoskeletal diseases, e.g., rheumatoid arthritis, acute exacerbations of osteoarthritis, ankylosing spondylitis, and juvenile idiopathic arthritis [17,18]. The drug is available as tablets for oral administration and as intramuscular injections. Meloxicam is usually used in a daily single dose of 15 mg. Among patients with a high risk of side effects, treatment is started with a dose of 7.5 mg and then increased to 15 mg [19,20]. 

Many drugs have been shown to have a high affinity for melanin biopolymers. Long-term pharmacotherapy with MLX may cause drug accumulation in the skin tissues. It may result in drug retention in pigmented cells, where small, non-toxic doses of the medication may be released gradually from melanin complexes. The greatest risk is the accumulation of the drug in melanin biopolymers, which may result in a significant increase in the concentration of MLX in the superficial skin tissues, which may cause a higher risk of the phototoxic effect occurrence [21].

There have been many recent attempts to develop MLX formulations (liposomes, nanoparticles, and microemulsions) that could be applied topically and overcome the stratum corneum barrier. These dosage forms may have several benefits related to the reduction of common side effects, such as gastrointestinal bleeding, headaches, and an increased risk of cardiovascular events. Even though the transdermal administration of meloxicam may be a promising alternative to conventional delivery pathways of NSAIDs, it can also be associated with the problem of intensifying other side effects such as phototoxicity [22,23,24,25]. Currently, available meloxicam formulations are issued as both a prescription and OTC medicines, and similar to other NSAIDs, they have many adverse reactions that occur during pharmacotherapy, including phototoxicity [26]. 

Due to emerging reports about the phototoxic potential of meloxicam, the purpose of this study was to evaluate the influence of MLX and UVAR on homeostasis of human normal skin cells–dermal fibroblasts (HDF) and epidermal melanocytes (HEMn-LP).

## 2. Results

### 2.1. The Estimation of Normal Human Skin Cells Proliferation after Exposure to Meloxicam and UVA Radiation

**Figure 1:**
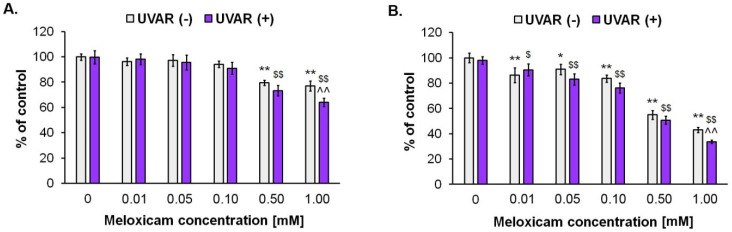
The impact of MLX and/or UVAR on the fibroblasts (**A**) and melanocytes; (**B**). The cells were pretreated with MLX at concentrations ranging from 0.01 mM to 1 mM and subsequently exposed to UVAR. The results are presented as a cell number ratio. * p < 0.05 vs. untreated cells (control), ** p < 0.01 vs. untreated cells (control), $ p < 0.05 vs. irradiated cells (not treated with meloxicam), $$ p < 0.01 vs. irradiated cells (not treated with meloxicam), ^^ p < 0.01 vs. corresponding sample not treated with UVAR.

An introductory assessment of meloxicam and UVARs antiproliferative influence on human skin cell proliferation was made using Cell Proliferation Reagent WST-1. The effects presented in Figure 1 suggest that MLX inhibits cell proliferation in both cell lines, and UVAR contributes to an additional reduction in cell proliferation. In the fibroblasts, MLX and UVAR reduced cell numbers by approximately about 20% and 30% at 0.5 mM and 1 mM, respectively. In melanocytes, co-treatment with MLX and UVAR reduced proliferation in a wider range of concentrations—from 0.01 mM to 1 mM. The greatest effect was observed in concentrations of 0.5 mM and 1 mM, at about 50% and 60%, respectively. The phototoxic effect of MLX on both cell lines was observed, especially at concentrations of 0.5 mM and 1 mM; therefore, it was decided to conduct further studies using these drug concentrations. 

### 2.2. The Evaluation of Cell Number and Viability of Human Normal Skin Cells Treated with Meloxicam and Exposed to UVA Radiation

The estimation of human normal skin cell number and viability was conducted using the fluorescence image cytometer NucleoCounter® NC-3000™ (ChemoMetec, Lillerød, Denmark). 

**Figure 2:**
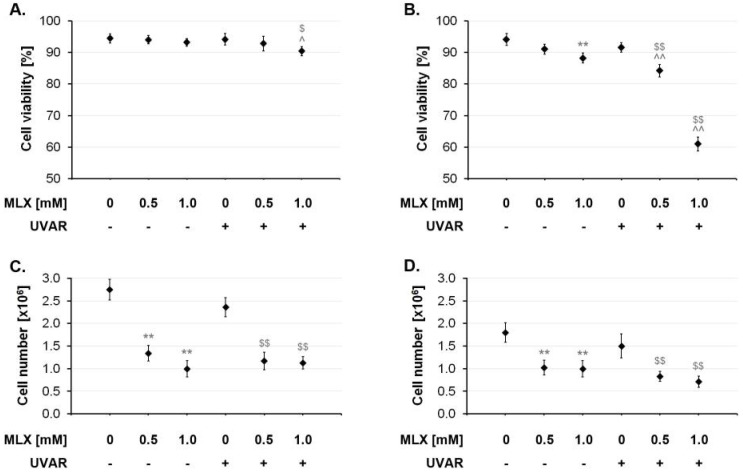
The influence of MLX and/or UVAR on fibroblasts (**A**,**C**) and melanocytes (**B**,**D**) viability and cell number. The cells were pretreated with MLX at concentrations 0.5 mM and 1 mM and then exposed to UVAR. ** p < 0.01 vs. untreated cells (control), $ p < 0.05 vs. irradiated cells (not treated with meloxicam), $$ p < 0.01 vs. irradiated cells (not treated with meloxicam), ^ p < 0.05 vs. corresponding sample not treated with UVAR, ^^ p < 0.01 vs. corresponding sample not treated with UVAR.

The data presented in Figure 2 showed that MLX had a different effect on cell viability depending on the cell line being treated. For both cell lines, the use of UVA radiation did not affect cell viability. A significant decrease in cell viability was observed for fibroblasts only at the co-treatment with MLX at a concentration of 1 mM and UVA radiation. There was a difference between the decline effect in the viability of melanocytes treated with the drug alone and MLX combined with UVAR in the concentration of 1 mM (approx. 8% vs. approx. 30%). 

The UVA radiation alone did not significantly reduce the number of fibroblasts statistically. MLX at a concentration of 0.5 mM and 1 mM caused a reduction in the total cell population by 50% and 64%, respectively. The combination of MLX and UVA radiation reduced the number of cells at both concentrations by about 50% compared to the irradiated assay. A cell count assay carried out on the melanocytes showed that the tested drug at both concentrations reduced the total cell population by about 45%. The effect of co-treatment with MLX and UVAR decreased the total number of cells by about 54% at the highest MLX concentration compared to the irradiated assay. 

### 2.3. The Evaluation of Morphology of Human Normal Skin Cells Treated with Meloxicam and Exposed to UVA Radiation

**Figure 3:**
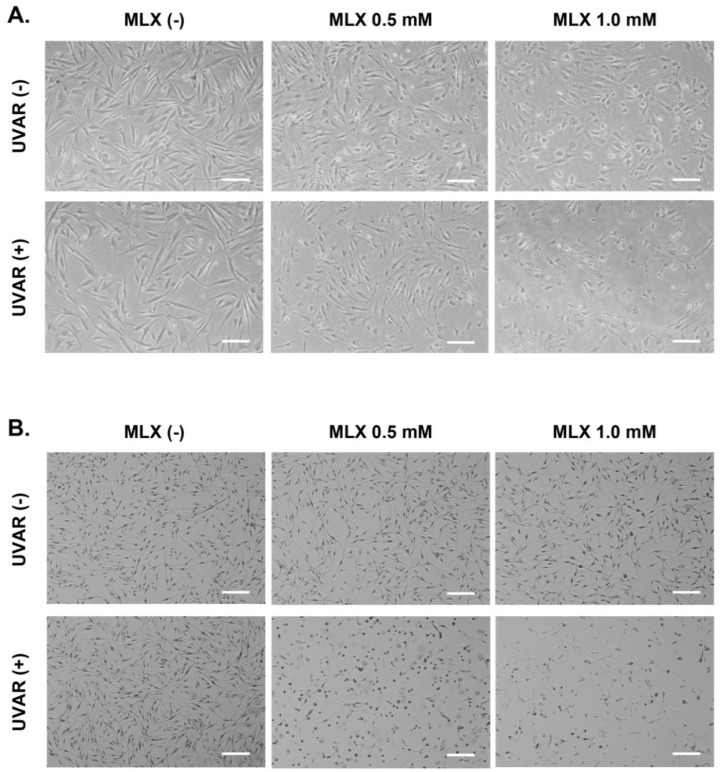
The impact of MLX and/or UVAR on the morphology of fibroblasts (**A**) and melanocytes (**B**). The cells were preincubated with MLX for 24 h, irradiated with UVAR and post-incubated with drug-free medium (scale bar = 200 µM).

The effect of MLX and/or UVAR on cell culture morphology was observed by the use of an inverted microscope, Eclipse TS-100-F (Nikon, Tokyo, Japan). As demonstrated in Figure 3**,** both types of normal skin cells differ mainly due to the presence of melanin pigment. Fibroblasts are more transparent without clearly marked cell nuclei. Due to the presence of melanin, melanocytes have better visibility under the microscope. In addition, melanocytes have a spindle shape and protrusions. According to the photos, it can be concluded that UVA radiation itself did not affect the shape, number or morphology of cells in both cell lines. MLX in both cell lines did not enhance the cytotoxic effect of the tested drug. The most noticeable cell morphology disorders were caused by simultaneous exposure to MLX and UVAR. UVA radiation revealed the phototoxic effect of meloxicam and caused a significant reduction in the percentage of cells with regular morphology, leading to an increase in the number of spherical cells without connections between adjacent cells. 

### 2.4. Analysis of Mitochondrial Transmembrane Potential in HDF and HEMn-LP Skin Cells Treated with Meloxicam and UVA Radiation

To detect changes in mitrochondrial transmembrane potential (ΔΨm) in skin cells treated with MLX and/or UVAR staining with JC-1 dye was conducted. JC-1 stain has the ability to accumulate in mitochondria in a potential-dependent way; in healthy cells with polarized mitochondria, JC-1 is located in the matrix of the mitochondrium and exhibits red fluorescence, while in cells with depolarized mitochondria it accumulates in the cytoplasm and exhibits green fluorescence. 

**Figure 4:**
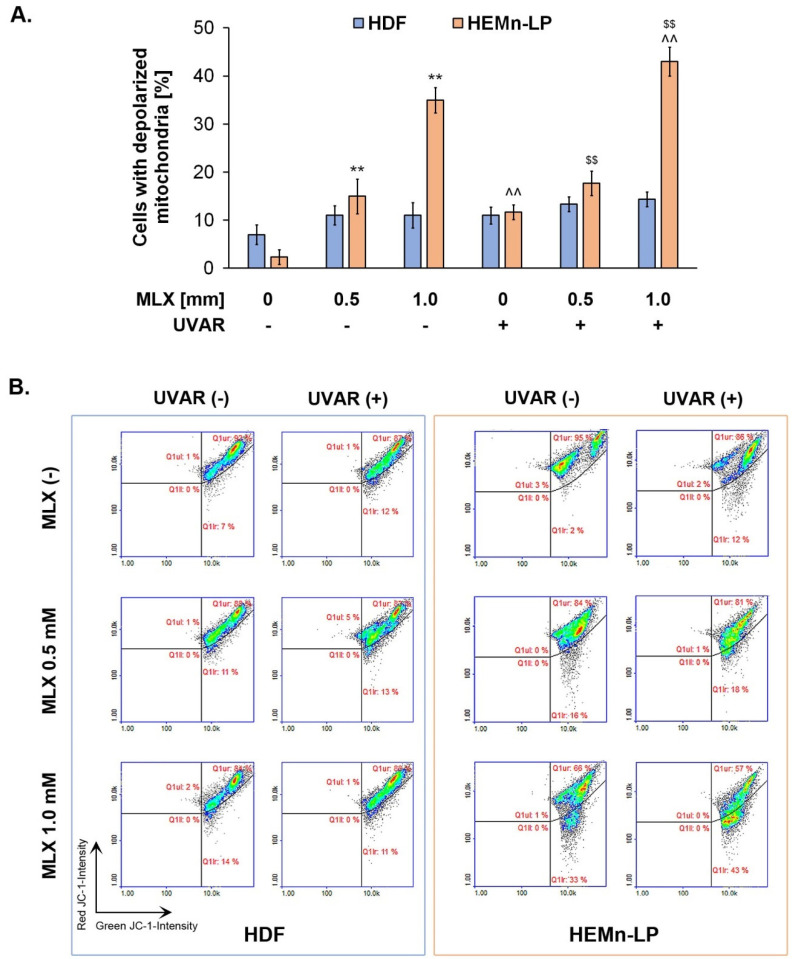
The changes in mitochondrial transmembrane potential in fibroblasts and melanocytes after treatment with MLX and/or UVAR. (**A**) Bar graphic representing cells with depolarized mitochondria; (**B**) Representative scatter plots presenting the changes in JC-1 density in analyzed cell cultures. ** p < 0.01 vs. untreated cells (control), $$ p < 0.01 vs. irradiated cells (not treated with meloxicam), ^^ p < 0.01 vs. corresponding sample not treated with UVAR.

The obtained results presented in Figure 4 showed that neither UVR exposure did not affect the mitochondrial potential in fibroblasts, nor treatment with MLX alone and co-treated with UVAR (approx. 12% vs. 11% in the concentration of 1 mM), which proves that the studied drug did not induce oxidative stress dependent on the mitochondrial pathway. 

In contrast, melanocytes turned out to be more sensitive to the effects of meloxicam and UVAR in terms of changes in the transmembrane mitochondrial potential. MLX induced a dose-dependent increase in the percentage of melanocytes that have depolarized mitochondria at concentrations of 0.5 mM and 1 mM by approximately 15% and 30%, respectively. Co-treatment with MLX and UVAR revealed that UVA radiation causes an additional increase in the percentage of cells with depolarized mitochondrial potential (ca. 34% with MLX, but without UVAR vs ca. 45% with MLX and UVAR in concentration of 1 mM).

### 2.5. The Influence of Meloxicam and Co-Treatment with Meloxicam and UVA Radiation on Cell Cycle

Cell cycle evaluation in fibroblasts and melanocytes was made by using image fluorescent cytometry. The influence of MLX on human normal skin cells seems to be varied depending on the type of cells that differ in the presence of melanin pigment. 

**Figure 5:**
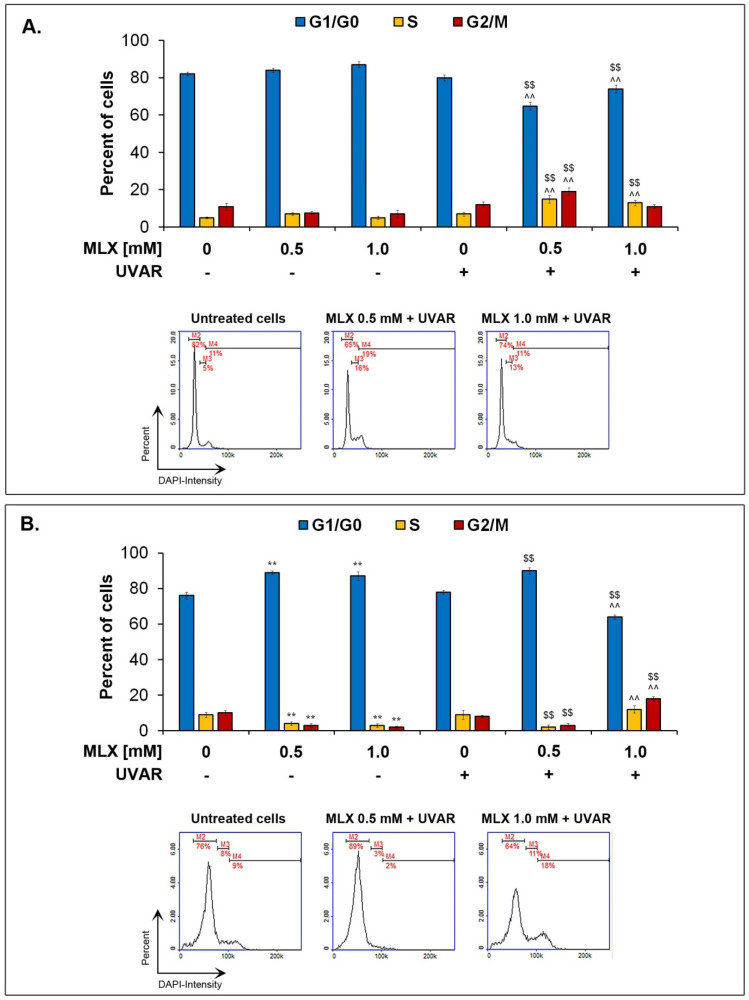
Bar graphics represent the number of cells in a particular cell cycle phases after treatment with MLX and/or UVAR on fibroblasts (**A**). The impact of MLX and/or UVAR on cell cycle on melanocytes (**B**). ** p < 0.01 vs untreated cells (control), $$ p < 0.01 vs irradiated cells (not treated with meloxicam), ^^ p < 0.01 vs corresponding sample not treated with UVAR.

Obtained results presented in Figure 5 indicate that both agents—UVA radiation and MLX used separately did not affect the cell cycle profile in fibroblasts. Co-treatment with MLX and UVAR causes changes in the cell cycle at concentrations of 0.5 mM and 1 mM that lead to a decrease in the percentage of cells in the G1/G0 phase by about 15% and 5%, respectively, and an increase in the percentage of cells in the S phase by approximately 10% and 6%. Additionally, a concentration of 0.5 mM increased the number of cells in the G2/M phase by about 10% was observed.

A significant influence of MLX and UVAR on the melanocyte cell cycle profile was noted. MLX causes cell cycle arrest in the G1/G0 phase. Data showed an increase in the percentage of cells in the G1/G0 phase by 13% and 8%, respectively, at concentrations of 0.5 mM and 1 mM, and a decrease in cell level in the S and G2/M phases by about 5% at both concentrations. MLX at a lower concentration combined with UVA radiation maintains a tendency to increase the percentage of cells in the G1/G0 phase by about 25% and decreases in the S and G2/M phases by approximately 8%, which was the evidence of a cell cycle arrest occurrence. The highest concentration of MLX in combination with UVA radiation decreased the percentage of cells in the G1/G0 phase by about 10%, and the percentage of cells in the G2/M phase increased by approximately 8%. The obtained results suggest that MLX at a concentration of 1 mM used in combination with UVA radiation induces the cell cycle arrest in the G2/M phase. The phototoxic effect of meloxicam was revealed during the use of the drug at the highest concentration on the melanocytes.

### 2.6. Meloxicam Depreciates Normal Human Skin Cells Vitality by Decreasing Reduced Thiols Level 

**Figure 6:**
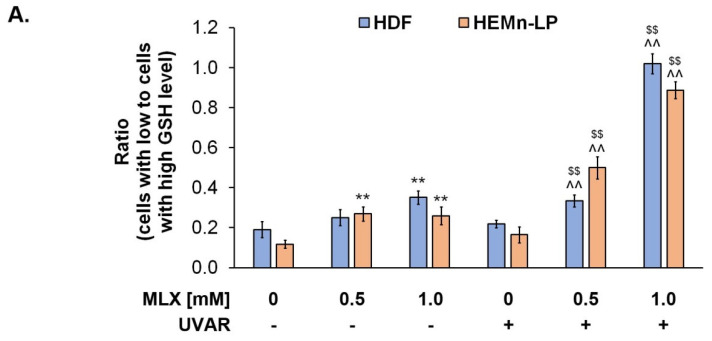

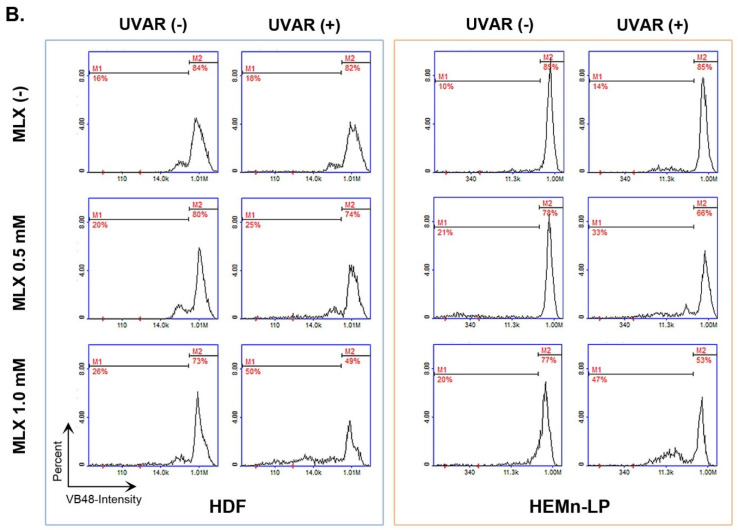
The evaluation of MLX and/or UVAR on the GSH level of fibroblasts and melanocytes. Obtained results are presented as a relative ratio of cells with low to cells with high GSH levels (**A**). The cells were pretreated with MLX at concentrations 0.5 mM and 1 mM, subsequently exposed to UVAR, and post-incubated in the medium for 24 h; (**B**) Representative histograms presenting the changes in the content of reduced thiols in cells treated with MLX and co-treated with MLX in concentrations of 0.5 mM and 1 mM and UVAR. ** p < 0.01 vs untreated cells (control), $$ p < 0.01 vs irradiated cells (not treated with meloxicam), ^^ p < 0.01 vs corresponding sample not treated with UVAR.

The analysis of intracellular thiol levels was prepared for MLX using a specific VitaBright-48™ reagent, which has the ability to combine with reduced thiols, i.e., glutathione, resulting in the formation of a fluorescent product. The obtained results, presented in Figure 6, indicate that MLX depreciates the level of reduced thiols. 

The UVA exposure did not affect the skin cells’ vitality in both cell lines. The observed percentage of fibroblasts with low GSH levels increased during the treatment with meloxicam in a dose-dependent way—a concentration of 1 mM increased the percentage of low-vitality cells by about 0.2. Simultaneous exposure of fibroblasts to MLX and UVAR caused an additional increase in the percentage of cells with low GSH levels—about 0.2 for meloxicam at a concentration of 0.5 mM and approx. 0.8 for a concentration of 1 mM was observed, compared to the control.

In the case of melanocytes, an increase in the percentage of cells with low GSH levels by approximately 0.2 for both concentrations can be observed. Meloxicam used in melanocytes did not increase the consumption of reduced thiols in a dose-dependent way. This effect was only observed during co-treatment with MLX and UVA radiation. The effect of an increased percentage of melanocytes with reduced thiol levels was approximately 0.3 and 0.7, respectively, for MLX at concentrations of 0.5 mM and 1 mM. 

The obtained results indicate that meloxicam caused the phototoxic effect and depreciated redox homeostasis in human normal skin cells.

### 2.7. Redox Homeostasis of Human Normal Skin Cells Exposed to Meloxicam and UVA Radiation

Phototoxic reactions are associated with intracellular ROS generation, which is the symptom of the occurrence of oxidative stress that causes disturbances in redox homeostasis and inflammatory reaction induction. The intracellular level of ROS was assessed using the H_2_DCFDA assay.

**Figure 7:**
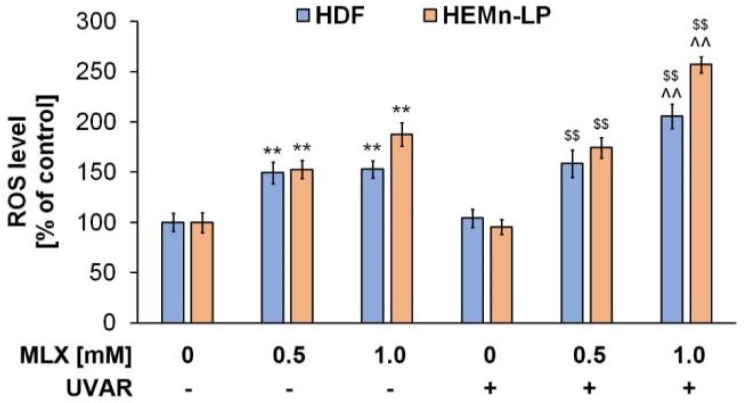
Redox homeostasis in HDF and HEMn-LP cell lines exposed to MLX and/or UVAR. The cells were treated with MLX at concentrations of 0.5 mM and 1 mM for 24h and exposed to UVAR. H_2_DCFDA assay was conducted to determine the intracellular ROS level. Obtained results were calculated as the percentage of control. ** p < 0.01 vs untreated cells (control), $$ p < 0.01 vs irradiated cells (not treated with meloxicam), ^^ p < 0.01 vs corresponding sample not treated with UVAR.

To determine the impact of MLX and/or UVAR on the oxidative-reduction homeostasis of human normal skin cells, the quantified ROS levels in HDF and HEMn-LP cell lines were measured (Figure 7). The exposure of HDF cells to MLX caused a statistically significant increase in the percentage of ROS, but co-treatment with MLX and UVA radiation caused an additional increase in ROS levels in normal skin cells (approx. 50% vs. approx. 100%, respectively).

The obtained data suggest that in the melanocytes, treatment with meloxicam increased the ROS level by about 75% for the concentration of 1 mM. Co-treatment with MLX and UVAR enhanced the effect induced by the drug alone and led to an increase in the ROS level of about 150% at higher concentrations.

### 2.8. Meloxicam-Melanin Complex Formation

**Figure 8:**
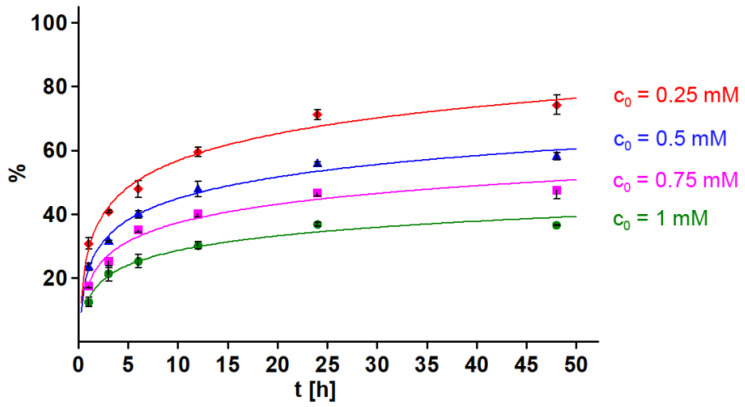
Effect of incubation time and initial meloxicam concentration (C_0_) on the amount of drug bound to DOPA-melanin (in %).

Meloxicam forms complexes with DOPA-melanin, and the amount of the drug bound to the biopolymer increases with the increase in the initial concentration of the drug and with the extension of the complexation time, reaching equilibrium after approx. 24 h (Figure 8).

## 3. Discussion

Due to their analgesic, anti-inflammatory and antipyretic properties, NSAIDs are one of the most popular medicines, which were confirmed in the WHO’s Model List of Essential Medicines. Escalation of musculoskeletal problems makes NSAIDs usage unavoidable [15]. 

Drug-induced photosensitivity can be described as an adverse cutaneous response to the simultaneous usage of photosensitive agents and exposure to UV radiation [5]. Many drugs from the NSAIDs group, such as ketoprofen, ibuprofen, diclofenac, or piroxicam are widely known as phototoxic drugs [27]. However, the mechanism of meloxicam’s potentially phototoxic properties on skin cells has not been elucidated. The assessment of the risk of damage to skin cells exposed to meloxicam and UVA radiation seems to be of great importance in view of the similarity in the chemical structure of meloxicam and piroxicam as well as the development of new drug formulations for application directly to the skin. 

The skin is an organ made up of many different types of cells, including melanin-producing cells called melanocytes, which play a key role in skin protection by the ability to absorb UV radiation. The process of pigment production is initiated in melanocytes to protect DNA against mutations caused by UVR. Melanin biopolymers are produced by L-tyrosine enzymatic transformation to dopaquinone. Biochemical reactions subsequently lead to oligomers formation, 5,6-dihydroxyindole-2-carboxylic acid (DHICA) and 5,6-dihydroxyindole (DHI), which are basic constituents of pheomelanin and eumelanin [27,28]. Melanin also shows cell-protective properties due to the ability to chelate metal ions, scavenge ROS, or form complexes with exogenous substances such as drugs. Although the binding of substances to melanin is reversible, it may result in their retention within pigmented cells [29,30]. Thus, long-term pharmacotherapy with drugs showing the ability to accumulate in melanin-containing cells may cause, among other things, a reduction in the effectiveness of the treatment and an increase in the risk of ADRs, including phototoxic reactions. Simultaneously, the accumulation of drugs in melanocytes may evoke changes in the antioxidant properties of melanin and disrupt redox homeostasis in cells [30,31,32].

In the first stage of the study, the impact of MLX and UVAR on fibroblasts and melanocyte proliferation was assessed. Taking into consideration the obtained results, the antiproliferative effect induced by MLX and UVAR observed in the preliminary WST-1 screening assay was confirmed by the results of studies conducted on large cell populations, but it is mainly due to the cytotoxic activity of meloxicam. The greater antiproliferative effect of MLX and UVA radiation was obtained in fibroblasts, but all cells had high viability. A stronger effect induced by MLX and UVAR was observed on melanocytes because, in addition to the antiproliferative effect, the effect of reducing cell viability was also observed. 

Drugs from the NSAIDs group in combination with UVA radiation reduced the proliferation of both normal and neoplastic cells, depending on the concentration and dose of radiation used. Previously conducted studies by Banach et al. showed that another drug from the NSAIDs group—ketoprofen combined with UVAR—also reduced the number of normal skin cells [33]. Other studies indicated that UVAR with drugs from the NSAIDs group—acetylsalicylic acid, ketoprofen, and ibuprofen also reduced the number of neoplastic cells, e.g., MCF-7, MDA-MB-231, or COLO829 [33,34,35]. It was proven that minocycline and other tetracycline antibiotics and lomefloxacine, moxifloxacine and other fluoroquinolones used both irradiated and in combination with UVAR reduced the proliferation of melanocytes [1,36,37]. Among the mentioned drugs, both tetracyclines and fluoroquinolones have a proven strong ability to bind to melanin and accumulate in pigmented cells. Moreover, the drugs show strong cytotoxic and phototoxic properties. The obtained results suggest that melanin plays a key role also in phototoxicity, as melanocytes turned out to be more sensitive to the phototoxic drug action than fibroblasts, which do not contain melanin biopolymers. It is worth emphasizing that the role of melanin and drug accumulation in pigmented cells in the mechanism of development of phototoxicity was also confirmed for other drugs from the NSAIDs group, e.g., ketoprofen [33]. 

Additionally, the obtained microscopic images of normal skin cells confirmed the results obtained in preliminary studies—WST-1 and cell count and vitality assay. 

Due to the demonstrated antiproliferative activity of meloxicam, in the next stage, the analysis of the cell cycle was performed. The cell cycle consists of the interphase, which includes the G1, S and G2 phases, and the mitotic (M) phase. During interphase, the cell prepares for mitosis (M-phase) by growth and DNA replication. The M phase begins with mitosis, resulting in reproduction through the cell cycle, resulting in genetically identical daughter cells [38,39,40,41]. Based on the conducted research, it can be concluded that meloxicam affects the cell cycle of normal skin cells depending on the concentration and the cell line tested. The phototoxic effect of meloxicam was associated with a deregulation of the cell cycle in the HDF line simultaneously exposed to UVA radiation. In melanocytes considered to be more sensitive to the effects of meloxicam, the effect of the drug itself on the course of the cell cycle is apparent. UVA radiation induces additional changes, deepening the effects of MLX. 

The analysis of cell cycle distribution carried out on the following three bladder cancer cell lines: HT1376 T24 and 5637 and on HepG2 hepatocellular cancer cell line after treatment with meloxicam showed similar effects compared to the normal melanocyte line [41,42]. The arrest of the cell cycle in the G1/G0 phase was observed in both bladder cancer cells, liver cancer and melanocytes. The resulting disturbances in the course of the cell cycle confirm the inhibitory effect of MLX on cell proliferation by limiting their ability to continue cell division through the accumulation of cells in the G1/G0 phase.

Drug-induced phototoxicity is directly connected with oxidative stress occurrence [9]. Significantly increased ROS production, especially superoxide anion radicals (·O^-^_2_), singlet oxygen (^1^O_2_), hydroxyl radicals (·OH) and hydrogen peroxide (H_2_O_2_), is the cause of intracellular damage within the nucleic acids [43]. Oxidative damage to DNA can result in gene mutations, which may consequently lead to complete cell dysfunction, resulting in the induction of carcinogenesis or apoptosis [43,44,45]. Mitochondria may be partly responsible for the growth of ROS levels. Mitochondrial dysfunction caused by NSAIDs is connected with mitochondrial electron transport chain complex-I and may lead to activation of harmful redox chain reactions, followed by a bioenergy crisis, and result in cell death [14]. Free electrons generated during mitochondrial dysfunction cause a partial reduction of molecular oxygen and thus generate reactive oxygen species, which are able to perturb the intracellular redox homeostasis. The resulting ROS are able to functionally inactivate cellular macromolecules such as DNA, proteins or lipids and can lead to the activation of mitochondrial depolarization [45,46]. Based on previously conducted studies of NSAIDs, it was found that drugs of this group, including meloxicam, are factors that have a harmful impact on the functioning of mitochondria [47].

The results indicate that MLX reduces the mitochondrial transmembrane potential in the melanocytes in a dose-dependent effect, and the UVA radiation deepens the effect. Based on the obtained results, it can be concluded that in the case of melanocytes, oxidative stress may be activated resulting from mitochondrial dysfunction. In contrast, the slight effect of lowering the transmembrane mitochondrial potential in the HDF cell line may be due to the different characteristics of both cell lines, i.e., melanin content, which suggests a different mechanism of oxidative stress prevention. 

Reduced glutathione (GSH) is a peptide composed of the following three components: cysteine, glycine and L-glutamate, located mainly in the cytosol and in small quantities in organelles, i.e., the nucleus, mitochondria and reticulum [48]. The main role of GSH as the most frequent intracellular antioxidant is the neutralization of harmful free radicals and maintaining redox homeostasis within the cell [49,50]. The exposure of melanocytes and fibroblasts to MLX causes the consumption of antioxidant compounds, which suggests that in the skin cells, reactive oxygen radicals have been generated. Additional exposure to UVR increased the percentage of cells with a low content of reduced thiols, which proves the phototoxicity of the drug. The studies conducted in rat gastric tissue indicate that MLX significantly reduced the content of free glutathione in the cells and was an inducer of the generation of free oxygen radicals, which confirms the results obtained with the use of skin cells [51,52,53]. 

The obtained data indicate that meloxicam alone induces ROS generation in both lines of normal skin cells. However, simultaneous exposure of HDF and HEMn-LP cells to UVAR and MLX caused the greatest increase in ROS levels, suggesting that the possible mechanism of the phototoxic action of meloxicam is based on the photodynamic reaction.

The study showed that MLX in combination with UVAR in a dose-response relationship significantly decreases the level of reduced thiols and increases ROS levels in both cell types, which leads to the formation of numerous oxidative cell damage. Concluding, meloxicam in combination with UVA radiation has a stronger effect on cells with melanin, which may be associated with the formation of drug-melanin complexes, accumulation of MLX in these cells and the achievement of higher concentrations in them. Due to the processes carried out by melanocytes related to the biosynthesis of melanin pigments, these cells are characterized by an increased content of free oxygen radicals and a reduced basic activity of antioxidant enzymes, which additionally predisposes cells to the phototoxic and oxidative damage of cell organelles. Due to the multitude of cells and structures of the skin that are exposed to UV radiation, it is necessary to conduct further studies to test the full phototoxic effect of meloxicam. Therefore, oral and intramuscular intake of meloxicam, similar to novel topical preparations such as gels, patches or creams applied directly to the exposed UVR skin, can be hazardous from a photosensitizing point of view.

## 4. Materials and Methods

### 4.1. Chemicals and Reagents

Meloxicam was obtained from Boehringer Ingelheim (Budapest, Hungary). Fibroblasts Growth Medium, amphotericin B solution (250 µg/mL), penicillin, phosphate-buffered saline (PBS) and L-3,4-dihydroxyphenylalanine (L-DOPA) were purchased from Sigma Aldrich Inc. (St. Louis, MO, USA). Neomycin sulfate was acquired from Amara (Kraków, Poland). An M-254 growth medium and a human melanocyte growth supplement-2 (HMGS-2) were obtained from Cascade Biologics (Portland, OR, USA). Trypsin/EDTA was obtained from Cytogen (Zgierz, Poland). Via-1-Cassettes™ (acridine orange and DAPI fluorophores), NC-slides A2 and A8, as well as Solution 3 (1 µg/mL DAPI, 0.1% triton X-100 in PBS), Solution 5 (VB-48™ PI AO), Solution 7 (200 µg/mL JC-1) and Solution 8 (1 µg/mL DAPI in PBS) were purchased from ChemoMetec (Lillerød, Denmark). H_2_DCFDA reagent was acquired from Thermo Fisher Scientific Inc. (Waltham, MA, USA). WST-1 cell proliferation reagent was obtained from Roche GmbH (Mannheim, Germany). Other chemicals were obtained from POCH S.A. (Gliwice, Poland).

### 4.2. Cell Culture

In vitro studies were performed on human dermal fibroblasts (HDF) and human epidermal melanocytes (HEMn-LP), which were purchased from Cascade Biologics (Portland, OR, USA). All cells used in the research were from passages 5–10. HDF cells were cultured in Fibroblasts Growth Medium. The HEMn-LP cells were cultured in the growth medium M-254, which was supplemented with HMGS-2 as well as antibiotics, as follows: amphotericin B (0.25 µg/mL), penicillin (100 µg/mL), and neomycin sulfate (10 µg/mL). 

### 4.3. Cells Treatment and UVA Exposure

Fibroblasts and melanocytes were seeded into a 96-well microplate (5000 cells/well) and into Petri dishes (7.5 × 10^5^ cells/dish). The cells were preincubated in the appropriate growth medium at 5% CO_2_ humidity and 37 °C. The medium was replaced by meloxicam solutions after 48 h of incubation. At that time control samples were cultured in the growth medium. The medium/drug solutions were changed after 24 h for PBS. The cells with PBS were exposed to UVA radiation (ʎ_max_ = 365 nm) using a filtered lamp BVL—8.LM (Vilber Lourmat, Eberhardzell, Germany). The dose of UVAR was 5 J/cm^2^. At the same time, the non-irritated cells had been kept in PBS in dark conditions at 37 °C and 5% CO_2_. After the incubation time, PBS was removed, and cells were incubated in the appropriate medium until analysis. 

### 4.4. Cells Proliferation Screening Assay

The proliferation of normal skin cells varied by the melanin content was estimated by WST-1 colorimetric assay. In brief, WST-1 (4-[3-(4-iodophenyl)-2-(4-nitrophenyl)-2H-5-tetrazoilo]-1,3-benzenedisulphonate) is a tetrazolinum salt, which is transformed to formazan by metabolically active cellular mitochondrial dehydrogenases. The amount of obtained product correlates with the number of viable cells. All cell types were seeded in a 96-well microplate (5 × 10^4^ cells/well) in a Fibroblasts Growth Medium and M-254 medium, respectively. Cell cultures were incubated at 37 °C and 5% CO_2_ for 48 h. After the incubation period, the medium was replaced by meloxicam solutions in the concentration range from 0.01 mM to 1 mM. After 24 h the medium was replaced by DPBS and cells were exposed to UVA radiation. After 21 h of incubation 10 µL of WST-1 reagent was added to each well and the incubations were continued for another 3 h. The absorbance was measured at 440 nm with a reference wavelength of 650 nm using the microplate reader Infinite 200 Pro controlled by the Magellan software. The viability of the skin cells was expressed as the percentage of untreated control cells.

### 4.5. Cell Number and Cell Viability Assay

Cell viability and abundance were assessed by using an imaging cytometer NucleoCounter® NC-3000™. The analysis is based on detecting dead cells by using DAPI stain and total cells population with acridine orange stain. Cell cultures were harvested 24 h after UVA irradiation exposure, centrifuged, and resuspended in the PBS. Then, they were loaded into Via1-Cassettes™ containing stains. The cells were analyzed by Cell Viability and Cell Count Assay by an NC-3000™ cytometer. DAPI is a dye that penetrated the permeable cell membrane, which allows determining the percentage of the population of non-viable cells.

### 4.6. Cell Morphology Assessment

Photographic documentation of HDF and HEMn-LP cell morphology characteristics under the influence of meloxicam was carried out during the experiment. Cells were seeded in Petri dishes (7.5 × 10^5^ cells/dish) and cultured in an appropriate growth medium, as previously described. The treatment with meloxicam in concentrations of 0.5 mM and 1 mM began 48 h after seeding. Then the drug solutions were replaced by DPBS and cells were exposed to UVAR. The cell culture was examined under a light inverted microscope NIKON TS100F.

### 4.7. Intracellular Thiols Level Analysis

The estimation of the intracellular thiols level was performed by the use of the fluorescence imaging cytometer NucleoCounter® NC-3000™. The assay is based on a specific dye–VitaBright 48™ which selects cells with a high level of reduced thiols, e.g., GSH. The treatment with meloxicam began 24 h after reaching a logarithmic growth phase. After 24 h the drug solutions were replaced by PBS and exposed to UVAR. After the exposure period cells were incubated with a non-drug medium. The cells were harvested by trypsinization and suspended in an appropriate growth medium in an amount of 1 × 10^6^ cells/mL. Subsequently, 10 µL of Solution 5 was added to 190 µL of cell suspension. The stained cells were loaded into 8-chamber NC-Slides A8™ and analyzed using the “vitality (VB-48) assay” protocol in the fluorescent image cytometer NC-3000. The obtained results in the form of VB-48™ histograms were used to differentiate cells subpopulations with varied content of reduced thiols and indicate health status–high level of reduced thiols (healthy cells).

### 4.8. Mitochondrial Potential Assay

The modifications in mitochondrial transmembrane potential (ΔΨm) were indicated by using the fluorescence image cytometer NucleoCounter® NC-3000™. The analysis is based on the red fluorescent cationic dye JC-1, which accumulates in the mitochondria in healthy cells. In cells with reduced mitochondrial potential, the green, fluorescent JC-1 dye tends to be localized in the cell cytoplasm. Human normal skin cells were seeded in Petri dishes (7.5 × 10^5^ cells/dish) and cultured in an appropriate growth medium until reaching the logarithmic growth phase. Then the cells were treated with meloxicam in 0.5 mM and 1 mM concentrations at 24 h and exposed to UVAR in PBS. After the UVA exposition period, the cells were incubated in a drug-free medium until the analysis was performed. Sequentially, the cells were harvested by trypsinization and 12.5 µL of Solution 7 was added to 1 × 10^6^ cells and incubated for 10 min at 37 °C. Afterward, the stained cells were centrifuged at 400× *g* for 5 min and washed twice with PBS. Obtained cell sediment was resuspended in 0.25 mL Solution 8 and analyzed using NC-Slide A8 and Mitochondrial Potential Assay protocol. The percentage of cells with low mitochondrial potential was presented on obtained scatter plots.

### 4.9. Cell Cycle Analysis

The cell cycle analysis of both HDF and HEMn-LP cells was performed using an imaging cytometer NucleoCounter® NC-3000™. The assay is based on the DNA content measurements, which investigate the cells’ population. Briefly, the cells were seeded at Petri dishes (7.5 × 10^5^ cells/dish), after 48 h were treated with meloxicam solutions. After 24 h treatment, the drug solutions were replaced by DPBS, and cells were exposed to UVAR and post-incubated with a drug-free medium for 24 h. Before the analysis cells were trypsinized, suspended in PBS (1 × 10^6^ cells/mL), and fixed with 70% cold ethanol. The cells were stained with Solution 3, loaded into NC-Slide A8 and analyzed by using the Fixed Cell Cycle DAPI protocol. The different phases of the cell cycle in tested cell cultures were demarcated basis on the obtained DNA content histograms.

### 4.10. H_2_DCFDA Assay—Quantyfing ROS Level Analysis

The H_2_DCFDA test was used to determine the level of ROS in human normal skin cells (HDF and HEMn-LP) after treatment with MLX and UVAR. Briefly, cells were seeded in 96-well dark microplate (5 × 10^3^ cells/well) in an appropriate growth medium for 48 h. After the incubation period, the medium was removed for MLX solutions in the concentrations of 0.5 mM and 1 mM. Subsequently, the cells were irradiated with UVA and post-incubated with a medium without the drug for 24 h. The H_2_DCFDA reagent was added to cells and incubated for 30 min in the darkness, and washed twice with PBS solution. The microplate reader Infinite 200 Pro was used to measure the fluorescence intensity (ʎ_em_ = 530 nm; ʎ_max_ = 485 nm). The results were normalized to the cell number and expressed as a control percentage.

### 4.11. Melanin Synthesis

Synthetic DOPA-melanin was prepared according to the model methodology described by Binns et al. [54]. Oxidative polymerization of L-DOPA prepared in phosphate buffer (0.067 M; pH 8.0) was carried out for 48 h.

### 4.12. Meloxicam–Melanin Complex Formation

Meloxicam complexes with synthetic melanin were performed by incubation of 5 mg melanin samples with 5 mL drug solutions. In the study meloxicam solutions ranging from 0.25 mM to 1.0 mM were used. Simultaneously, control samples with distilled water were prepared. All samples after incubation period at room temperature were filtered. 

### 4.13. Analysis of Meloxicam Binding to Melanin

The spectrophotometric method was used to quantify the analyzed drug. The amount of melanin-bound drug was determined as the difference between the amount of drug introduced into complexation and the amount of unbound drug determined in the supernatant. Measurements were carried out using a JASCO V-630 UV-VIS spectrophotometer (Tokyo, Japan) at a wavelength of 271 nm (ε_271_ = 10108 dm^3^ × mol^−1^ × cm^-1^).

### 4.14. Kinetics of Drug-Melanin Complex Formation

The complexing kinetics of meloxicam with melanin was assessed on the basis of the relationship between the amount of drug bound to the polymer, complexation time and the initial drug concentration. The following incubation times were used in the research: 1 h, 3 h, 6 h, 12 h, 24 h, 48 h and drug concentrations: 0.25 mM, 0.5 mM, 0.75 mM, 1.0 mM.

### 4.15. Statistical Analysis

In all experiments, mean values of at least three separate experiments performed in triplicate (*n* = 9) ± standard deviation (SD) were calculated. Statistical analysis was performed using GraphPad Prism 7. Differences among groups were assessed using one-way ANOVA analysis of variance followed by Dunnett’s test; *p* < 0.05 was determined to indicate a significant difference.

## Data Availability

Not applicable.
